# Supplementary material for: Psychosocial hazard exposures and mental health outcomes among ambulance Emergency Medical Technicians in Ghana: A qualitative phenomenological study
Source: PLOS Ment Health. 2026 Mar 30;3(3):e0000483. doi: 10.1371/journal.pmen.0000483 (PMC13035119; doi:10.1371/journal.pmen.0000483)
Supplement: S1 Table — (PDF) [file pmen.0000483.s001.pdf]

S1 Table: Thematic framework for psychosocial hazard exposures in Ghana's National Ambulance Service

Data Codes	Main theme	Subthemes	Definitions	Example Quotes
**Psychosocial hazard exposures**	Factors perceived as causes or sources of psychosocial hazards EMTs are mostly exposed to			
	Bearing the Burden	The physical, cognitive, emotional, and ethical demands of ambulance work		
		Physical demands	The physical exertion and strain required in the job including lifting heavy objects, providing first aid, and transporting patients	"We have risk with our ear, being on board for a long time with the siren, you stand the risk of having ear problem. When you are moving a patient maybe when we get to the hospital you have risk of injuring your spine in moving a patients who are heavy, you can hurt yourself. We have some patients they are very heavy, when you are moving that patient and you do not move them well, we call it lifting so, if you don't lift them well you might injure your spine. So, we have all this risks." EMT2<br>"We might also develop spinal injury with time because we lift patients, the more you are getting pressure on the spine with time you are going to be getting spinal problems, that is back pains, waist pain different conditions that affect the spine. A colleague for instance was lifting a patient and all he could hear was a sound at the back and it affected him a lot. At a point, he couldn't walk well without support, these are the types of hazards we face."
		Risk of infection, injury and death	Potential dangers faced by EMTs, including the risk of contracting diseases, sustaining physical injuries, and even facing life-threatening situations	"The risks are many. There is travelling risk that is the movement of the ambulance, and it can crash with other vehicles. We have so many instances where our ambulances crash and we lose our members; we have those risks. And then infection risk, when you get to accident scene and you are not careful, you are likely to get infected because you come in contact with fresh blood and then anything can happen." EMT2<br>"Our job setting involves lots of risk and some patients even transfer infections to us in the course of treating them." EMT6<br>"You know this our portholes road [bad road network], could make you hit any part of your body on a hardware. Some patients are very agitated, and you need to be standing with all this. Some of our colleagues have been involved in an accident and a lady died and apart from that in the night too armed robbers when they hear our siren, I don't know whether they think we are police, they also attack us" EMT11

Data Codes	Main theme	Subthemes	Definitions	Example Quotes
		Cognitive demand	Mental challenges and cognitive workload experienced by EMTs, such as making critical decisions quickly, assessing patient conditions, and effectively managing emergencies	"Sometimes you even forget that you are an EMT. I remember there was a mass casualty case … So as the incident commander, I quickly organized some people and with the limited resources, we managed to save all lives. …So yes sometimes when you are at such chaotic scenes, you get confused and we sometimes seek for help from other ambulance stations as a backup." EMT11<br>"Sometimes when you get to the scene and realized your patients are so many that they outnumber the EMTs at the scene, instantly, you become overwhelmed and confused of where to start from with the limited resources so sometimes you have to call for backups. There come the victims who become agitated, with lots of shouts here and there expressing pains. Sometimes you even have some standbys there telling you what to do, when the scene is chaotic, you will see human parts scattered or separated with blood everywhere. All these makes you confused on the field and when you get back, psychologically you are not yourself, you begin to have nightmares, you can't even sleep or eat."<br>"So you have to work as soon as possible to resuscitate the patient before you get to the hospital that's why immediately you have to stop everything …so at that moment you don't have time to relax" EMT3
		Emotional demand	Emotional toll and stress experienced due to exposure to traumatic situations, witnessing suffering, and dealing with intense emotions while providing care	"Road traffic accident which involves so many people dying. So, after that, you become so much devastated and then you break down because you see lives lost." EMT2<br>"You become traumatized, especially when the case is very bad, you've been able to move the patient because the patient could die on the ambulance, but you've been able to manage the case to the hospital. And then upon arrival or at the triage unit, you lose the patient, you become more traumatized"
		Work overload	Being burdened with excessive work demands, leading to a high volume of calls, extended work hours, and limited time for rest and recovery	'By now, even as we are talking, I have to perform at least two or three roles at the office right now, after coming back from the scene, or whatever we went to do. Now before you alone will sit down and write, once you have your tablet, you have to enter everything into the tablet, mostly, sometimes you don't have adequate time to relax.' EMT13

Data Codes	Main theme	Subthemes	Definitions	Example Quotes
				"I am a regional administrative manager, most of the time you have a lot of reports to submit. You have quarterly reports, monthly reports, other incidents, mid-year reports, managing people, every aspect of the work in the region. Like yesterday, when you called, you saw the time I was leaving the office. And I still had a lot of things to do. Even, when I was leaving, I have to take some of my files home," EMT10
		Moral injury	Having to care for a victim while not having appropriate resources/conflict between professional and ethical values on the one hand and responders' own safety or the availability of resources on the other.	'You can become more frustrated because you would be like you suffered so much to bring the patient to the hospital. Therefore, you feel for the patient and feel like if the patient should have died at where you picked him/her that would have been better. So it will be like all your efforts have been in vain and you become more frustrated. And sometimes you would be thinking maybe there is something you should have done, you didn't do and you've lost the patient.'EMT2<br>'Even sometimes, some week's koraa you can think about some cases you attended that you think you shouldn't have lost the patient. You wonder what you did wrong that made you lose the patient. What should you have done that could have made the patient survive?'EMT14
		Inadequate work-life balance	Challenge of balancing work responsibilities with personal life commitments, which can lead to stress, fatigue, and difficulties in maintaining a healthy lifestyle	'…sometimes when you want to spend time with your family, a call can come in and take almost 3 hours to 8 hours of your time and before you get back your family is asleep' EMT11<br>'… I am affected in such a way that I come home, I am unable to sleep and the next day, you want me to wake up early, iron the kids uniform, help with the children's homework, help my wife; I won't be able to do them because it's like I am tired. The woman won't understand and she may think like you are not helping her and she will be complaining. So it's like what you're supposed to do at home, you won't be able to do because you're tired, you're worried, unable to sleep, you wake up but you still feel tired. You know when you go through stress, it comes with all these things. You're still thinking about things and when you are asked to help, you are not ready because there are a lot of things you are thinking about. You go to bed, you are asked to do certain things, you are unable to do it because it becomes very difficult for you. In fact, it can affect you family life' EMT10

Data Codes	Main theme	Subthemes	Definitions	Example Quotes
	Systemic Strain	The organizational, infrastructural, and resource challenges that create operational barriers for EMTs		
		Unconducive employment conditions	Unfavourable work environment, including inadequate facilities, and suboptimal work conditions that hinder EMTs effectiveness.	"You know the work we do is very dangerous and with regards to transport, you see the speed at which we drive and anything can happen. And you have a family you have left at the house, do you know that upon all this that we're doing, we don't receive any risk allowance. So if there is any accident there is no allowance for you or your family." EMT1<br>"Financially, I would say our salary is very bad. Imagine even me as a senior person I'm at level 18 and I started 12 years ago and I was placed at level 16. I've gone on two promotions. When you have your qualification in other areas [health sectors], you qualify to be a director and all that. Your starting point is even deputy director. So you can imagine, you completed your masters years ago, …your colleagues elsewhere are directors and you are still at level 18 when they are at level 24, 25 and all that. What they take [salary], yours is not near them. So, it's shameful the way we are treated' EMT10
		Logistical constraints	Challenges faced in terms of limited access to certain resources such as PPE shortages, 'No bed' syndrome, understaffing,	"..in Ghana our fuelling system the ambulance is bad. The vehicles are fuelled by the government and before you are given the fuel coupons, you must go through a lot of channels. Can you imagine that our ambulance can be there for two months, the ambulance can be out of commission due to shortage of fuel? So when there is an emergency or a severe case and they need your help you can't move the ambulance. And you can't do anything about it." EMT1<br>"Most of the places too, we don't even have offices of our own. So we are actually perching with other institutions. At times, what to work with in the office, logistics becomes a challenge. There are some places, if you want to submit your report, computers and all that and even tables and chairs to sit on is a problem at some places'EMT10<br>'You must have all your consumable sets, PPE and what have you but at times they are not up to the standards." EMT11<br>"…sometimes we experience shortage of gloves but it is the responsibility of the government to supply us all these things. And sometimes we have to ask for gloves from the hospital when we go for referral cases which is not the best. For the tools you have to disinfect

Data Codes	Main theme	Subthemes	Definitions	Example Quotes
				it and use the same tools for the next patient which is not good but due to shortage, that is the only way to go" EMT1
				"…anytime we are being dispatched to rescue accident victims, the control calls the receiving hospital and informs them that we are bringing accident victims from this jurisdiction. But by the time we get there they will tell us there is no bed and in such instances we have to wait for the patient to be transferred to the bed which can take like 5hours which is not good; looking at urgency of the case. So if there is a new accident case that I have to attend to and the ambulance is stationery at the hospital because of no bed, I can't go and handle any case again." EMT1<br>"The level of cooperation from hospital staff is good but at times there is a problem of no beds. There is a patient you have transported, they've given you all the details, you called, no bed, wait. Sometimes, this no-bed issue prolongs and before you realise, the patient is expired, dead in your ambulance." EMT10
	Between Stigma and Support	The social context within which EMTs operate		
		Insufficient Organizational and Public Support	Can be supportive and unsupportive. Overall atmosphere and psychological environment in the workplace, including factors such as job satisfaction, organizational culture, and employee wellbeing	"The support we get most often is fuel support... but for one month everything is finished within a week, and you have about 3 weeks to the end of the month" (EMT10)<br>"The public is never ready to even give us fuel support to support severe cases. At times the public doesn't understand why they would call, and we would say there is no fuel." (EMT11)<br>"The government is not supportive so mostly the patient's relatives complain about why we are charging, while the government is telling the public that ambulance operation is free" (EMT13)<br>"Unfortunately, as a service I'm yet to see any psychological unit that will psych us up after such chaotic scenes. During training we were taught when we come back, we have something called debriefing. We sit and psych ourselves as a form of evaluation." (EMT 11)
		Relationship conflict	Disagreements, tensions, or difficulties in interpersonal relationships between EMTs,	"..sometime we shout at each other when we get there. There is no seniority.., everybody is under pressure. Ideally, our driver is always supposed to be in the car so when we are ready and he is no where to be found, that is where we get angry and shout at each other and at

Data Codes	Main theme	Subthemes	Definitions	Example Quotes
			colleagues, supervisors, or other stakeholders, which can negatively impact teamwork and job satisfaction	times because of the limited resources, we are confused at the scene, and it always takes the best of us. We just reconcile ourselves, after then we move on." EMT11<br>"Sometimes where two of you are overwhelmed, you begin to shout at each other which at times leads to fights and hatred. But after everything is done, we reconcile with each other. This has ever happened to me. I was transporting a case with my junior colleague and she got overwhelmed and didn't even know how to connect an oxygen to the patient and I got angry and said to her that how can she claim she is saving life but can't fix an oxygen. At that moment I didn't know what I was doing she become cold to me so after 3 days time, I realized I was wrong so I had to apologize to her and we reconciled" EMT6
		Stigmatisation and violence	Social stigma and potential for encountering violent situations that EMTs may face due to the nature of their work	"And then when you pick patients which are very aggressive, they injure you by that act of aggressiveness, if you do not handle the patient well by strapping the patient well or tie their hands to the trolley. And then if you move their hand; they can injure you through the process. So, we have all those risks, I call it aggressive patients who are unstable." EMT2<br>"Sometimes we have others risks like when you are attending to patients and relatives are there and they feel like…you are not doing what you're supposed to, or you are not being fast for them. They tend to quarrel with you or maybe attack you, so we stand those risks and sometimes they feel like our response was low and they attack us with insults." EMT2<br>"Other health workers at the hospital level... when we transport victims to the hospital, on arrival the nurses turn to harass us that we kept long, forgetting the distances and the nature of the road... they also think we don't have to rush them to take over the case. At times, these nurses do not value the services of the EMT... they think they are superior to EMTs, so they don't take any contributions we make towards the survival of our victims. Some of them talk to EMTs with no respect, but others who know and value our services talk to us with respect. I have received more than 3 complaints from EMT drivers that nurses insult them." EMT6

Data Codes	Main theme	Subthemes	Definitions	Example Quotes
		Poor coordination with receiving facility	Lack of effective coordination and collaboration among different stakeholders involved in emergency response, which can lead to delays, confusion, and compromised patient care	…sometimes when we get to the receiving facility, they say they did not called, sometimes handling over becomes a problem especially, when we reach Korle Bu, they call before we start the journey but when you reach there they said the doctor they called is not on duty so they are not aware of the case, now you have to wait for them or they reject the case or they ask you to try different facility. You know it's a big challenge, it's a traumatized situation.' EMT12<br>'Even the hospital staff who should understand the system but no, their back and forth and arguments with EMTs delays victims for hours and at times, they will force us to keep the patients in our ambulance and sometimes the patient dies in the process; oh it is a whole lot.' EMT11
		Bystander disruptions	Interference or disruptive behaviour from bystanders at the scene of emergencies, which can hinder the efforts of EMTs and compromise the safety of patients and responders	'I am talking about one of the problems like you've been dispatched to an accident scene to take patients to the hospital but by the time you reach there, the bystanders would have taken the victims away. But, they are not trained, they don't know anything. By the time your reach there, you'd not see anybody there.' EMT1
		Negative public perception	Negative attitudes, misperceptions, or lack of appreciation from the public towards EMTs	'…the perception of ambulance not good. A lot of people think that we carry corpse. So when they ask you what work do you do and you tell them you work with ambulance service, they begin to withdraw from you some even become afraid of you' EMT6
		Poor recognition and praise	Lack of acknowledgment or appreciation for the efforts and contributions of EMTs	'Oh at times when we get to the scene, especially if it mass casualty, even the police are afraid of the blood. At that instance, we are more or less a saviour. Everybody gives us that respect though apart from other recalcitrant citizens who may impede our operations they always give us the chance to work so I feel always happy. Yeah, that people have confidence in me; in us that we are coming to save life.' EMT11<br>'..I don't think the public recognizes us for what we do. When we go for cases and come back, we only get 20% recognition of our services. Only few people recognize our efforts and appreciate us.' EMT6
**Impacts of psychosocial**	Effects of being exposed to psychosocial hazards in emergency response			
		Detrimental impacts of psychosocial hazards exposure experiences		

**Data Codes**	**Main theme**	**Subthemes**	**Definitions**	**Example Quotes**
**hazard exposures**	Psycho-emotional distress	Vicarious trauma	Psychological and emotional impact experienced by EMTs as a result of repeated exposure to traumatic events, leading to symptoms similar to posttraumatic stress disorder (PTSD).	'I get nightmares at times. There was a burnt at the scene and you could see the body roasted and all that. And you get traumatized.' EMT7<br>'…at times when you go to the field of an RTA case, and then you see people's intestines, everywhere is full of people crashed, you see human parts all spread around, you do your best at the scene, and you think everything is okay but when you come home, it will take you about a whole week, you won't be able to eat well. You'll be thinking about it. At times it directly or indirectly affects you. You won't even realise that but before you realise it's disturbing you psychologically' EMT10
		Fatigue/Mental Exhaustion	State of physical and mental exhaustion due to long working hours, demanding schedules, and the cumulative effects of sleep deprivation	'…you know the body itself is also a machine and when you over work it out it gets out of hand sometimes, when you work and work and work at a point you get too much stressed out and even forget what you are supposed to do because of tiredness.'EMT3<br>"it's too much when the cases keep coming, because sometimes you can respond to three or four cases because of the stressful nature of the job you would become worn out and wish you have some time to rest." EMT3
		Depression	Mood disorder characterized by persistent feelings of sadness, hopelessness, and a lack of interest or pleasure in activities, which can be triggered or exacerbated by the stressful nature of the work and the experiences encountered	'.. I sometimes get emotionally disturbed and depressed at such scenes even to the point that it affects my thinking, feeling and ability to move around work successfully and also get confused.<br>I remember I had to go the hospital for checkup because I felt all was not well with me and after investigation, everything turned out to be negative, and I realize it was depression'.. EMT6
		Work rumination	Tendency of ambulance EMTs to continuously think about work-related issues, events, or traumatic experiences even when	'That's when you lost a patient, the whole day and even, when you get to the house, you can still think about it. If not that God has created forgetfulness, we wouldn't be able to forget some of the cases. Even sometimes, some weeks koraa you can think about some cases you attend that you think you shouldn't have lost the patient.…….so you

Data Codes	Main theme	Subthemes	Definitions	Example Quotes
			off-duty, leading to persistent preoccupation and difficulties in mentally disengaging from work	think through all the day maybe two days you will be thinking about the case.' EMT2<br>'Sometimes I get flashbacks to the point that I get home and I am still thinking about it and it then it turns to affect my sleep. I sometimes get scared that even the person who died could visit me' EMT1
		Maladaptive coping	Adoption of unhealthy ways to manage traumatic experiences	"We have people who are addicted to alcohol [heavy drunkards]. Yes, and some of them, they unable to stop. We advise them, we talk to them. We try to transfer them but even some don't have the money to relocate to their new stations and you have to allow them to stay. They are so broke so you have to just consider them. There was a time I had three serious drunkards at one station and the CEO came and was like why have you put all these guys here? You need to separate them," EMT 10<br>"Because of time delays, a victim can pass on at your care. Some of the scenes we witness are terrible and horrible, so at times I have to take in alcohol to even have appetite for food." EMT 13
	Positive outcomes	How participants have developed resilience and positive growth despite the negative experiences		
		Post-traumatic growth	Positive psychological changes and personal growth after exposure to traumatic events, such as increased resilience, enhanced personal relationships, and a greater appreciation for self and life, higher sense of accomplishment and understanding of the work.	'…it has changed my personal life in the positive… it made me see that this world is not as people see it in terms of my comportment, I relate freely with everyone regardless of my position in the office. If I don't tell you I'm the superior you won't know because I don't see human being as anything. No matter who you are, when I remember the calibre of people who become helpless, motionless, paralysed, people cut into pieces, … all has made me see life from different perspective.' EMT11<br>'I appreciate life more and I understand being of human and then how to value life or how to approach situations' EMT2

Data Codes	Main theme	Subthemes	Definitions	Example Quotes
		Adaptive coping	The ability of EMTs to effectively manage and adapt to stress, adversity, and trauma, demonstrating resilience and psychological wellbeing in the face of challenging circumstances	'…personally I have psyched myself already these things are bound to happen so when it happens you have to psyche yourself up and move on provided it doesn't really cause any serious damage, even if it cause any serious damage I know it is bound to happen, psychologically it won't affect me that much. Someone who has not psyched himself up would definitely have a bit of psychological problem.' EMT3<br>'I will say, if I'm psychologically down with those problems, what I do to help myself is I have some worship songs on my phone, that I believe once I'm listening to them my problems, so normally that's what I do' EMT4<br>"It takes the grace of God to do this work. So, those who are not capable, we send them to administration." EMT6<br>"I called on God and God responded. I got to the facility and the doctor asked how I managed to, …, I said, it's by the Grace of God. And he said he did not understand, I said it's by the grace of God. Because myself I don't even know how I managed this case from Jasikan [a district town] till I reach…" EMT4<br>"If I'm psychologically down with those problems, what I do to help myself is I have some worship songs on my phone, that I believe once I'm listening to them my problems go away. So, normally that's what I do" EMT4
	Work attitudes	Positive and negative attitudes displayed by EMTs towards their work		
		Turnover intention	The likelihood to leave NAS for other organisations	'I have thought of leaving given a better opportunity, because I think I have been with the service for a long time and the level I should be is not where I am.'EMT6<br>'If I have any opportunity, I won't even look at what I am going to do actually. I'll look at how much am I going to get at the end of the month. And that will motivate me to leave or not to leave. How much am I going to get from there? I'll compare it to how much I am getting here. And if that place is better, I will leave; that one, I won't lie. At the end of the day, when you go on pension, whatever you are doing here it is fine but the rest of the life, you are going to live it' EMT10

Data Codes	Main theme	Subthemes	Definitions	Example Quotes
		Job satisfaction	Level of contentment and fulfilment experienced by EMTs in their work, influenced by work conditions, pay, recognition, support, and opportunities for professional growth.	'…for the job itself I am satisfied but the nature of the work and the salary, I am not satisfied. EMT2<br>'…I will say my satisfaction is like maybe fifty percent. My major worry is about our salary. It is very bad. So no matter how the job …. Whatever; but if pocket is not okay for you, it won't make you happy. That's the fact' EMT10
