# Supplementary material for: Psychosocial hazard exposures and mental health outcomes among ambulance Emergency Medical Technicians in Ghana: A qualitative phenomenological study
Source: PLOS Ment Health. 2026 Mar 30;3(3):e0000483. doi: 10.1371/journal.pmen.0000483 (PMC13035119; doi:10.1371/journal.pmen.0000483)
Supplement: S1 File — (PDF) [file pmen.0000483.s002.pdf]

Appendix 1. Interview Guide

Preamble/Informed Consent

This research is focused on the experiences of emergency responders in their work environment. I will be asking questions about how you manage to work to save lives during accidents, disasters and related emergencies, the types of demands such situations put on you and the resources available to you to cope with the situation. I will also be capturing issues related to how these experiences impact your emotions and mental state. Now, I am going to ask: ,.

- How long has it been since you responded to any emergency case?

Participants who qualify and are willing will be given an information sheet and a consent form to sign. It will be stressed that they are free to end the interview at any time if they so wish.

Section A: Personal Data/General Questions

1. What is your Job Title/Grade/Position?
2. How long have you been working at the National Ambulance Service?
3. Tell me about your job. What exactly do you do? What does your job entail?
4. Please what is your age or age range?
5. Level of education
6. Any training in emergency response?
7. Please share with me your marital status and number of dependents.

Section B: Subjective experience of Psychosocial Hazards

Please answer the following questions. Feel free to express yourself. I will be grateful if you explain your answers with examples where possible. Please avoid the use of actual names of individuals.

1. Tell me about your typical normal day on the job i.e., responding to an emergency.
2. Which kinds of emergencies have you handled?
3. Do you enjoy your job? What things about your job make it enjoyable?
4. What are some of the things about your work that you find to be challenging?
5. Tell me how you understand the term psychosocial hazards and risks at work?
6. Reflect on the following definition of psychosocial hazards and risks:

The adverse aspects of the work environment that pervade operations as a result of the structural characteristics of the job and the social life within the work context have the potential to harm your physical and mental health and well-being (Houdmont, 2013).

Based on this/your definition,

7. What things/factors will you consider psychosocial hazards and risks entail? Describe these factors.

8. What kinds of demands does your job place on you? Probe:
    a. Tell me about how much work you have to do. Shift schedules
    b. Are there times you are not sure what to do? Please explain.
    c. Do you have to think intensely in order to perform your task? Please explain
    d. Do you feel emotionally disturbed or drained? Please explain
    e. Do you get confused over how to approach a task between you and your colleagues? Please explain
    f. Do frictions with your colleagues occur due to differences in personality, values, and norms?
9. Narrate to me what kinds of resources are available to you. Probe:
    a. Are you able to work at your discretion and pace?
    b. Do you get support from your:
        i. Organisation i.e., risk allowance, insurance
        ii. Supervisor
        iii. co-worker –
    c. Do you get the necessary tools, implements and equipment to work with? Please Explain
    d. Do you feel recognised and praised for your work by the public? Please explain, Cooperation from hospital facility.
    e. Do you feel fairly treated in terms of policies, procedures and processes at work? Please explain.
    f. Are you adequately provided with information about changes in the workplace? Explain
10. Have you had any experience/s of being exposed to these things while working as an ambulance service personnel? Tell me more about this/these.

Section C: Subjective Experience during the Emergency Responses

11. In your emergency response related work, tell me the experiences you have had in relation to:
    a. How has the experience changed your impressions of this job?
    b. How do you personally experience the emergency scenes?
    c. What feelings did you have during the responses? Were there difficult situations? Were there good moments?
    d. Whether there were any effects on your mental state?
    e. Whether there were any effects on your personal or life values? Probe Positive effects/Perspective on life in general
    f. Whether it was distressing? Did it make you sad? How?

Section D: Impact of Emergency Response

12. Which thoughts or feelings do you have when leaving emergency scenes?
13. Do you leave work issues behind when you leave work?

14. There is the phenomenon that people who are in close contact with very stressed or traumatized people or those affected can also be psychologically burdened. Would you be able to say the same about yourself? What were the effects on your mental state?
    a. Probe instances of:
        i. Insomnia,
        ii. restlessness,
        iii. avoidance
        iv. re-experiencing,
        v. depressive symptoms,
        vi. anxiety (vicarious traumatization).
15. Have your values changed as a result of your previous emergency responses?
16. Explain the effects these experiences had on your personal and work attitudes? Probe any organisation-related consequences i.e., quit intention, employee engagement, organisational commitment, job satisfaction etc.

Section E: Processing of Experiences and Intervention

17. How do you manage the experiences you have during your response to an emergency or disaster situation? Probe:
    a. Have you talked to someone for psychosocial help? Did you discuss issues that you found distressing?
    b. What would you need to do to manage the experiences even better? Probe (Organisationally, In-service training, from supervisors, improvement of specific skills)
18. Do you feel supported in your work and emergency response?
    a. What support do you get?
    b. What is particularly important to you in your work or emergency response context?
    c. Do you feel valued for your work? Explain
    d. What kind of support would you like to receive? Probe (Organisationally, In-service training, from supervisors, improvement of specific skills).
19. What recommendations do have for improving ambulance emergency response in Ghana?
