# Supplementary material for: Psychosocial hazard exposures and mental health outcomes among ambulance Emergency Medical Technicians in Ghana: A qualitative phenomenological study
Source: PLOS Ment Health. 2026 Mar 30;3(3):e0000483. doi: 10.1371/journal.pmen.0000483 (PMC13035119; doi:10.1371/journal.pmen.0000483)
Supplement: Attachment — Submitted filename: Response to Reviewers.docx [file pmen.0000483.s005.docx]

**RESPONSE TO REVIEWERS**

**Manuscript ID**: PMEN-D-25-00415

**Title**: Psychosocial Hazard Exposures and Mental Health Outcomes Among Ambulance Emergency Medical Technicians in Ghana: A Qualitative Phenomenological Study

**Authors**: Elias Kekesi, Maxwell Asumeng, Ernest Darkwah and David Sam

**Date**: January 19, 2026

Dear Dr. Hanif Abdul Rahman and Reviewers,

We sincerely thank you for the opportunity to revise our manuscript and for the constructive feedback provided by the reviewers. We are pleased that all three reviewers found our manuscript technically sound and valuable. We have carefully addressed each comment and believe the revisions have significantly strengthened the manuscript.

Below, we provide a point-by-point response to each reviewer comment, indicating the changes made and their locations in the revised manuscript.

**JOURNAL REQUIREMENTS**

**Requirement 1: Inclusivity in Global Research Questionnaire**

**Response**: We have completed the Inclusivity in Global Research Questionnaire and included it as Supporting Information (S2 Questionnaire). We acknowledge that while our study was conducted by a Ghanaian researcher in Ghana with professional healthcare workers (rather than international research or community-based research), we have completed the questionnaire as requested. Most questions have been marked as “N/A” with appropriate explanations of why they do not apply to our study context.

**Requirement 2: Review of Suggested Citations**

**Response**: We have reviewed the citation suggested by Reviewer #1 regarding blockchain and machine learning for data management. We have incorporated this reference where appropriate (see response to Reviewer #1, Comment 5 and 6 below).

**Requirement 3: Reference List Review**

**Response**: We have thoroughly reviewed our reference list to ensure completeness and accuracy. We have verified that:

- All in-text citations match the reference list
- No retracted papers are cited
- All references follow PLOS formatting guidelines
- All URLs are functional and accessible

**REVIEWER #1**

We thank Reviewer #1 for the thorough and constructive feedback. All seven points have been addressed as detailed below.

**Comment 1:**

**Reviewer Comment**: “Ensure that the main outcomes in the abstract match those in the discussion section. The abstract mentions 'vicarious trauma, fatigue, and depression' as negative outcomes but does not include moral injury, which is a key finding discussed later. Consider adding 'moral injury' to the abstract to highlight this important finding”.

**Response**: We agree that moral injury is a critical finding that should be highlighted in the abstract. We have revised the abstract to include moral injury among the negative psychosocial outcomes.

**Comment 2:**

**Reviewer Comment**: “Rephrase the study aims into two or three clear research questions. For example: What are the lived experiences of psychosocial hazards for EMTs in Ghana's NAS? What mental health issues and work attitudes result from these experiences? How do cultural and resource constraints in Ghana affect EMTs' coping and well-being?”

**Response**: We appreciate this suggestion to improve clarity and focus. We have reframed our study aims as three clear research questions following the reviewer's suggested format.

**Revised text**: This study was guided by three research questions:

1. What are the lived experiences of psychosocial hazards among EMTs working in Ghana's National Ambulance Service?
2. What mental health outcomes and work attitudes result from these psychosocial hazard exposures?
3. How do cultural and resource constraints in Ghana's healthcare system influence EMTs' coping strategies and overall well-being?

**Comment 3:**

**Reviewer Comment**: “While the abstract and introduction summarize the study, the three specific aims in the Introduction (lines 387–391) are quite detailed. These should be framed more simply as clear research questions to guide the reader”.

**Response**: We have addressed this comment in conjunction with Comment 2 above.

**Comment 4:**

**Reviewer Comment**: “The current data availability statement is weak and may not meet PLOS journal requirements, which usually require full public access to a minimal dataset upon acceptance. Simply stating that raw transcripts will be available upon request is often not sufficient for open data policies.”

**Response**: We appreciate this important feedback regarding data availability. We have revised our Data Availability Statement to better align with PLOS open data policy while respecting participant confidentiality and ethical constraints.

**Comment 5:**

**Reviewer Comment**: “To strengthen the discussion on systemic failures and future healthcare data management, especially regarding potential fraud and decision-making in the National Ambulance Service (NAS), the authors should reference reliable advanced data processing research. A paper on ensemble machine learning for classifying blockchain transactional data is relevant since blockchain is mentioned in the study and could be a solution for transparent resource tracking.”

**Response**: We thank the reviewer for this valuable suggestion to strengthen our discussion of technological solutions for resource management challenges. We have incorporated a reference to advanced data processing and blockchain technology as a potential solution for transparent resource allocation in the NAS.

**Comment 6:**

**Reviewer Comment**: “In the Discussion under 'Systemic failures of chronic stressors' (or as a new paragraph in the Conclusion), add the following: 'Future strategies for managing the NAS's limited resources and preventing financial insecurity should explore secure and transparent data systems. Advanced machine learning models, such as ensemble learning, can accurately classify transactional data and could be used to monitor and secure resource allocation”.

**Response**: We have incorporated this important recommendation into our manuscript. We have added a new paragraph discussing future data management strategies using secure technological systems.

**Comment 7: Elaborate on Policy Recommendations Implementation**

**Reviewer Comment**: “The policy recommendations presented are good, but some, like 'mandatory patient handover protocols,' need more details on how they would be implemented in Ghana's National Ambulance Service (NAS) context to show their practicality”.

**Response**: We appreciate this feedback to enhance the practical applicability of our recommendations. We have expanded our policy recommendations section to include specific implementation details, contextual considerations, and practical steps for the Ghana NAS context.

**REVIEWER #2**

**Reviewer Comment**: “This was a very interesting and informative piece of research. Research on this topic is understudied, especially in West African countries. The research ended with good, practical recommendations for implementation.”

**Response**: We thank Reviewer #2 (Dr. Aylana Brewster) for the positive evaluation of our manuscript. We are pleased that the reviewer found our research interesting and our recommendations practical.

**REVIEWER #3**

**Reviewer Comment**: “This manuscript presents a well-executed qualitative phenomenological study examining psychosocial hazard exposures and mental health outcomes among ambulance emergency medical technicians (EMTs) in Ghana. It addresses a critical and underexplored area within global and occupational mental health, particularly in low- and middle-income country contexts, and aligns strongly with the aims and scope of PLOS Mental Health. The study design is well justified. The manuscript is well written, with clear structure and intelligent English. Overall, this is a high-quality and valuable contribution to the literature on emergency medical services and occupational mental health in resource-constrained settings. I have no substantive concerns and recommend acceptance of the manuscript as submitted.”

**Response**: We are grateful to Reviewer #3 (Dr. Sidra Batool) for this thorough and positive evaluation. We appreciate the recognition of our study's contribution to occupational mental health literature in low- and middle-income country contexts. The reviewer’s affirmation of our study design, writing quality, and overall contribution is encouraging and validates our research approach.

**Changes**: No specific changes requested.

**ADDITIONAL IMPROVEMENTS**

Beyond the specific reviewer comments, we have also:

- Proofread the entire manuscript for clarity and consistency
- Verified all participant quotes are properly anonymized
- Ensured consistent terminology throughout
- Checked all tables and figures for accuracy
- Verified that all ethical considerations are properly documented

We believe these revisions have significantly strengthened the manuscript and addressed all reviewer concerns. We are confident that the revised manuscript now meets PLOS Mental Health’s publication standards and makes an even stronger contribution to the literature on occupational mental health in emergency medical services.

Thank you again for the opportunity to revise and improve our manuscript. We look forward to your response.

Sincerely,

Elias Kekesi (Ph.D)

On behalf of all authors
